# How research on the meta-structure of psychopathology aids in understanding biological correlates of mood and anxiety disorders

**DOI:** 10.1186/2045-5380-2-13

**Published:** 2012-08-16

**Authors:** Shani Ofrat, Robert F Krueger

**Affiliations:** 1 University of Minnesota, 75 East River Road, Minneapolis, MN, USA

**Keywords:** Meta-structure, Comorbidity, Biological correlates, DSM-5

## Abstract

Research on biological correlates of psychopathology stands to benefit from being interwoven with an empirically based, quantitative model of mental disorders. Empirically-based classification approaches help to deal effectively with issues such as comorbidity among diagnoses, which often serve as challenges to interpreting research on biological correlates. With regard to the mood and anxiety disorders specifically, quantitative research shows how mood and anxiety disorders are well conceptualized as elements within a broad internalizing spectrum of psychopathology, such that many putative biological correlates of specific disorders may be better conceptualized as delineating the pathophysiology of the broader mechanisms underlying multiple disorders.

## Background

The stated goal of the Diagnostic and Statistical Manual for Mental Disorders (DSM) is threefold: to provide a helpful guide to clinical practice, to facilitate research and improve communication among clinicians and researchers, and to serve as an educational tool for teaching psychopathology (DSM-IV-R 4th ed., pp xxiii). Historically, DSM began as a broad set of vignettes that depicted common clinical manifestations of mental disorders, but diagnoses listed in early DSMs were not reliably diagnosed by clinicians, and as a result had little validity [1]. By introducing explicit sets of diagnostic criteria, the DSM-III was instrumental in refining the boundaries of diagnoses so that clinicians were discussing the same clinical entities, supposedly distinct from others. DSM-III also introduced exclusionary criteria that sought to limit overlap between diagnostic sets. DSM-IV did little to change the basic scheme of the DSM-III, which had grown to include hundreds of distinct diagnostic categories. This degree of presumed diagnostic differentiation often contrasts with clinical experiences, where patients typically meet criteria for multiple DSM diagnoses, a phenomenon referred to as comorbidity [2-6], or related experiences where patients present with symptom configurations “not otherwise specified”(NOS), as opposed to symptoms that cohere into a clear DSM-defined focal syndrome [2]. As a result, questions about the validity of DSM-IV’s approach to psychodiagnosis are frequently raised [1,7].

## Main text

**Figure 1:**
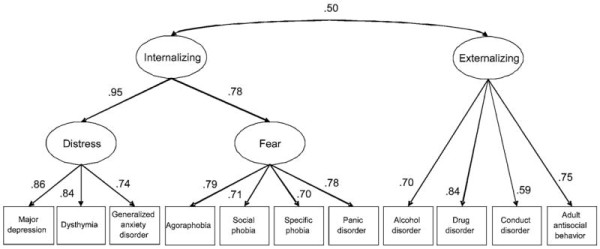
**Path diagram for best-fitting meta-analytic model of the structure of common mental disorders.** This figure provides parameter estimates from the best-fitting model according to a meta-analytic multiple-groups confirmatory factor analysis [9]. All parameter estimates are standardized and significant, p < .05. Reprinted from Krueger and Markon (2006). Copyright 2006 by Annual Review of Clinical Psychology. Reprinted with permission.

Research focusing on the problem of diagnostic comorbidity has resulted in a literature that has converged on an empirical model of the meta-structure of common mental disorders, or the way in which multiple mental disorders delineate spectrums of interrelated conditions. There is mounting evidence to suggest that common forms of psychopathology are defined by the higher-order groupings or spectrums of *internalizing* and *externalizing*, and that this shared underlying meta-structure contributes to the comorbidity among disorders [8-11]. As shown in Figure 1[9], internalizing is indicated by unipolar mood disorders and anxiety disorders, like major depressive disorder, generalized anxiety disorder, social and specific phobias, and panic, that load highly on the internalizing factor. Externalizing is indicated by disinhibitory and substance abuse disorders including drug, alcohol, and nicotine dependence, antisocial personality disorder and antisocial traits, pathological gambling, and conduct disorder. Within the higher-order internalizing dimension, some studies have revealed two sub-dimensions: distress, representing unipolar mood disorders and generalized anxiety, and fear, representing panic disorder, social and specific phobia [9], while others have not found evidence of these subfactors [12]. 

In expanding this work to encompass rarer forms of psychopathology, a recent study in a clinical sample analyzed data on 25 disorders, spanning both rarer and more common disorders, as well as Axes I and II. The best-fitting model consisted of 5 factors: (1) internalizing, which included anxiety and eating disorders, major depressive episodes, as well as cluster C (anxious and inhibited) personality disorders, borderline and paranoid personality disorders; (2) externalizing, including the substance use disorders and antisocial personality disorder; (3) thought disorder, including psychosis, mania, and cluster A personality disorders; (4) somatoform disorders; and (5) antagonism, including cluster B and paranoid personality disorders [13]. This work is important in that it replicates findings from previous work in a clinical sample and demonstrates that the same methods used to establish the existence of the internalizing-externalizing model can be used to extend that model to rarer disorders observed in clinical populations.

### Advantages of a quantitative-empirical approach over a case-control approach

As opposed to studies that take a meta-structure approach, studies that use a case–control design in looking for biological correlates of specific disorders are limited both conceptually and empirically in accommodating comorbidity among diagnoses. Searching for biological correlates of one specific disorder often involves designing research to identify cases that have no or few comorbid diagnoses, in order to maintain pure diagnostic categories. This artificial exclusion of cases with comorbidities means that the studies lose ecological validity, inasmuch as a typical clinical case will meet criteria for disorders in addition to the target disorder. Such studies are therefore difficult to extrapolate to actual clinical populations where there is extensive diagnostic comorbidity.

By contrast, in interpreting biological findings through the lens of psychopathological dimensions, research on the underlying biological substrates of disorders becomes more tractable because comorbidity is accommodated in study design and result interpretation. Disorders do not co-occur at random; instead, they group in predictable ways that the meta-structure helps us anticipate, and looking for correlates of these groups of disorders instead of singular diagnoses allows researchers to better reflect the realities of psychiatric diagnoses. Using the meta-structure approach in biological research also allows for a purer and stronger biological ‘signal’ by allowing disorders to co-vary rather than losing power to artificial distinctions between related diagnoses. The signal of the latent variable, or what is shared between diagnoses, will necessarily be stronger than the signal of each individual diagnosis, and will also reduce noise due to inter-diagnostic similarities that made it difficult to form conclusions about disorder specificity. Conversely, in the case–control designed study if comorbid diagnoses are allowed in the study design, it can be challenging to parse out the relationship between individual diagnoses and results with any sort of specificity, which is supposedly the benefit of conceptualizing disorders as separate entities.

Recent research on biological correlates of the internalizing spectrum illustrates these points. A recent review of studies investigating affect-startle in internalizing disorders found evidence that these particular biological correlates tend to line up with the two subfactors, i.e., fear and distress, that are frequently found to underlie the internalizing factor. Disorders marked by cue-specific fear (specific and social phobia) tend to be associated with one parameter of startle reactivity (potentiation of startle during exposure to aversive stimuli), whereas disorders marked by diffuse negative affectivity or distress tend to be associated with others (heightened general reactivity or increased context-potentiated startle and, possibly, decreased fear-potentiated startle). The authors conclude that there are specific features of fear-based reactivity that map onto the distress and fear sub-factors of internalizing, while the overall concept of pathological fear-based reactivity relates to the overarching internalizing spectrum, implicating fear-potentiated startle as a likely biological indicator of internalizing psychopathology generally [14]. The findings in this study are made more readily interpretable by the metastructure approach, which supports the distinction between fear and distress disorders under the internalizing factor. Additionally, this research demonstrates how research on biological underpinnings can also inform metastructure research itself by providing plausible mechanisms that can further clarify research on the ways in which disorders relate.

## Conclusions

The search for biological correlates of psychopathology stands to benefit from being interwoven with the empirical structure of psychopathology. Models of the empirical structure of psychopathology provide a coherent framework through which to interpret biological findings, which can accommodate comorbidity and improve ecological validity. It is important that subsequent versions of the DSM come to reflect the presence of the meta-structure underlying mental disorders if it aims to reflect our current empirical understanding of the data. A DSM-5 study group has proceeded towards the aim of representing the metastructure by developing a group of 11 ‘validating criteria’ that suggest aetiological relatedness between a cluster of disorders. The criteria are (1) genetic factors, (2) familiality, (3) early environmental adversity, (4) temperamental antecedents, (5) neural substrates, (6) biomarkers, (7) cognitive and emotional processing, (8) differences and similarities in symptomatology, (9) comorbidity, (10) course, and (11) treatment. A recent review of the literature has suggested that there is a case for the DSM to unify related disorders under the category of internalizing, also called emotional, disorders [15]. Although the meta-analysis found differences between disorders in the proposed cluster, specifically in the domains of cognitive and emotional processing, neural substrates, and familial studies, there were more similarities than differences in these disorders within the 11 domains studied. The authors suggest that a reorganization of the DSM to reflect this knowledge is possible, and further clarify that the proposed clustering would not remove traditional diagnostic categories, but only reorganize them to better reflect our current understanding. Although our understanding of etiology is still in its nascent stages, to preserve the current classification system with all its flaws until a definitive solution becomes clear would impede biologically and diagnostically-based research [16]. Insofar as the DSM 5 will reflect changes to our understanding of the nature of diagnostic categories, it will be progressing towards a system that will be better suited to research biological underpinnings.

## Competing interests

Neither author has any competing interests.

## Authors’ contributions

SO participated in drafting and editing the manuscript. RF Krueger participated in drafting and editing the manuscript. All authors read and approved the final manuscript.
